# Eukaryotic Organisms in Extreme Acidic Environments, the Río Tinto Case

**DOI:** 10.3390/life3030363

**Published:** 2013-07-04

**Authors:** Angeles Aguilera

**Affiliations:** Astrobiology Center, Spanish Institute for Aerospace Technologies, Carretera de Ajalvir Km 4, Torrejón de Ardoz, 28850 Madrid, Spain; E-Mail: aguileraba@cab.inta-csic.es; Tel.: +34-915-206-461; Fax: +34-915-201-064

**Keywords:** acidophiles, eukaryots, extremophiles, extreme environments, photosynthesis, Río Tinto

## Abstract

A major issue in microbial ecology is to identify the limits of life for growth and survival, and to understand the molecular mechanisms that define these limits. Thus, interest in the biodiversity and ecology of extreme environments has grown in recent years for several reasons. Some are basic and revolve around the idea that extreme environments are believed to reflect early Earth conditions. Others are related to the biotechnological potential of extremophiles. In this regard, the study of extremely acidic environments has become increasingly important since environmental acidity is often caused by microbial activity. Highly acidic environments are relatively scarce worldwide and are generally associated with volcanic activity or mining operations. For most acidic environments, low pH facilitates metal solubility, and therefore acidic waters tend to have high concentrations of heavy metals. However, highly acidic environments are usually inhabited by acidophilic and acidotolerant eukaryotic microorganisms such as algae, amoebas, ciliates, heliozoan and rotifers, not to mention filamentous fungi and yeasts. Here, we review the general trends concerning the diversity and ecophysiology of eukaryotic acidophilic microorganims, as well as summarize our latest results on this topic in one of the largest extreme acidic rivers, Río Tinto (SW, Spain).

## 1. Introduction

Our ongoing exploration of Earth has led to continued discoveries of life in environments that have been previously considered uninhabitable. For example, we find thriving communities in the boiling hot springs of Yellowstone, the frozen deserts of Antarctica, the concentrated sulfuric acid in acid-mine drainages, and the ionizing radiation fields in nuclear reactors [1,2,3,4]. We find some microbes that grow only in brine and require saturated salts to live, and we find others that grow in the deepest parts of the oceans and require 500 to 1,000 bars of hydrostatic pressure. Life has evolved strategies that allow it to survive even beyond the daunting physical and chemical limits to which it has adapted to grow. To survive, organisms can assume forms that enable them to withstand freezing, complete desiccation, starvation, high levels of radiation exposure, and other physical or chemical challenges. We need to identify the limits for growth and survival and to understand the molecular mechanisms that define these limits. Biochemical studies will also reveal inherent features of biomolecules and biopolymers that define the physico-chemical limits of life under extreme conditions. Broadening our knowledge both of the range of environments on Earth that are inhabitable by microbes and of their adaptation to these habitats will be critical for understanding how life might have established itself and survived.

## 2. Eukaryotic Extremophiles

When we think of extremophiles, prokaryotes come to mind first. Thomas Brock’s pioneering studies of extremophiles carried out in Yellowstone’s hydrothermal environments, set the focus of life in extreme environments on prokaryotes and their metabolisms [3]. However, eukaryotic microbial life may be found actively growing in almost any extreme condition where there is a source of energy to sustain it, with the only exception of high temperature (>70 °C) and the deep subsurface biosphere [4]. The development of molecular technologies and their application to microbial ecology has increased our knowledge of eukaryotic diversity in many different environments [5]. This is particularly relevant in extreme environments, generally more difficult to replicate in the laboratory.

Recent studies based on molecular ecology have demonstrated that eukaryotic organisms are exceedingly adaptable and not notably less so than the prokaryotes, although most habitats have not been sufficiently well explored for sound generalizations to be made. In fact, molecular analysis has also revealed novel protist genetic diversity in different extreme environments [4]. Temperature is one of the main factors determining the distribution and abundance of species due to its effects on enzymatic activities [6]. All extremophiles that survive at high temperatures (95–115 °C) are microorganisms from the archaeal or bacterial domains. On the contrary, for eukaryotic microorganisms, the highest temperature reported is 62 °C, and most of the metazoans are unable to grow above 50 °C [7].

Surprisingly, photosynthetic prokaryotes, such as cyanobacteria, have never been found in hot acidic aquatic systems [8]. Instead, these ecological niches are usually profusely colonized by species of the order *Cyanidiales*, red unicellular algae [8]. Thus, species from the genera *Galdieria* and *Cyanidium* have been isolated from hot sulfur springs, showing an optimal growth temperature of 45 °C and a maximum growth temperature of 57 °C [9,10]. These extreme hot springs are usually acidic (pH 0.05–4) and frequently characterized by high concentrations of metals such as cadmium, nickel, iron or arsenic, which are highly toxic to almost all known organisms.

Additionally, phototrophic eukaryotic microorganisms have colonized environments characterized by temperatures at or below 0 °C. Some algal species bloom at the snow surface during spring, and complex microbial communities have been found on glaciers, probably the most widely studies environments after marine ice habitats. Aplanospores of *Chlamydomonas nivalis* are frequently found in high-altitude, persistent snowfields where they are photosynthetically active despite cold temperatures and high levels of ultraviolet radiation [11]. Distinct microbial communities composed of psychrophilic bacteria, microalgae and protozoa colonize and grow in melt pools on the ice surface, or in brine channels in the sub-ice platelet in the Arctic even during winter, at extremely low temperatures of −20 °C [12].

Additionally, a number of heterotrophic eukaryotes have also been reported to inhabit extremely acidic environments. Thus, the yeast *Rhodotorula* spp. is frequently encountered in acid mine drainage waters, and isolates belonging to other genera (e.g., *Candida*, *Cryptococcusor Purpureocillium* sp*.*) have also been described [13,14,15]. Among the filamentous fungi that have been isolated from acidic sites, are some of the most acidophilic of all microorganisms: *Acontium cylatium*, *Trichosporon cerebriae* and a *Cephalosporium* sp. have all been reported to grow at ca. pH 0 [16].

## 3. Acidic Environments. The Río Tinto (SW, Spain) Case

Highly acidic environments are relatively scarce worldwide and are generally associated with volcanic activity and mining operation [17]. The natural oxidation and dissolution of the sulfidic minerals exposed to oxygen and water results in acid production, and the process can be greatly enhanced by microbial metabolism [1,18]. At the same time, low pH facilitates metal solubility in water, particularly cationic metals (such as aluminum and many heavy metals), and therefore acidic water tends to have high concentrations of heavy metals [19] (Table 1).

**Figure 1:**
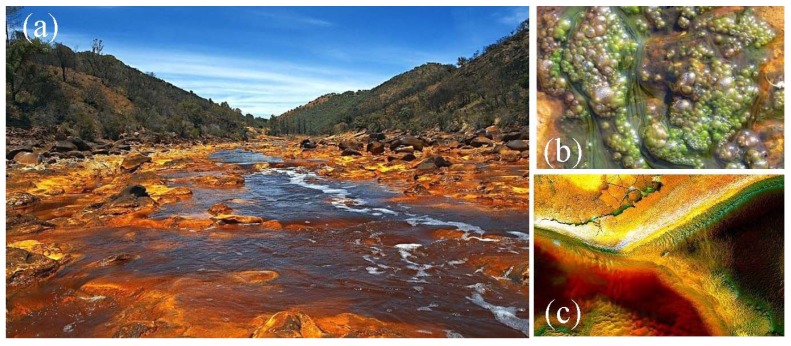
(**a**) General view of Río Tinto; (**b**) Photosynthetic biofilms formed by acidic *Klebsormidium* and *Zygnema*; (**c**) Photosynthetic biofilms formed by *Euglena mutabilis.*

Río Tinto (SW, Spain) is an unusual ecosystem due to its size (100 km long), rather constant acidic pH (mean value 2.3), high concentration of heavy metals (Fe, Cu, Zn, As, Mn, Cr, *etc*.) and high level of microbial diversity, mainly eukaryotic [20,21]. The river rises in Peña de Hierro, in the core of the Iberian Pyritic Belt, and reaches the Atlantic Ocean at Huelva (Figure 1). The Iberian Pyritic Belt is a geological entity of hydrothermal origin 250 km long and between 25 and 70 km wide, known to be one of the biggest deposits of metallic sulfides in the world [22,23]. One important characteristic of Río Tinto is the high concentration of ferric iron and sulfates found in its waters, products of the biooxidation of pyrite, the main mineral component of the system. Ferric iron is maintained in solution due to the acidic pH of the river and is responsible for the constant pH due to the buffer characteristics of this cation.

The combined use of conventional microbial ecology methods (enrichment cultures, isolation, phenotypic characterization) and molecular ecology, allowed most of the representative elements of the system to be identified. Eighty percent of the prokaryotic diversity in the water column corresponds to three bacterial genus: *Leptospirillum* spp., *Acidithiobacillus ferrooxidans* and *Acidiphilium* spp., all of them conspicuous members of the iron cycle [1].

**Table 1:** Physicochemical parameters at the most extreme sampling sites in Río Tinto (mean ± SD). Cond.—Conductivity (mS cm^−1^); Redox.—redox potential (mV). Ions in mg L^−1^ except Fe in g L^−1^.

Location	pH	Cond	Redox	Fe	Cu	As	Cd	Zn
Iz-Iz	1.8 ± 0.2	25.7 ± 2.3	569 ± 22	17 ± 4	12 ± 3	16 ± 4	43 ± 16	14 ± 3
ANG	1.5 ± 0.2	30.8 ± 3.4	471 ± 16	16 ± 3	132 ± 43	24 ± 3	30 ± 12	162 ± 5
UMA	1.6 ± 0.3	40.2 ± 8.3	473 ± 10	18 ± 7	85 ± 36	32 ± 5	40 ± 18	118 ± 4
RI	0.9 ± 0.3	38.9 ± 1.6	460 ± 30	22 ± 5	100 ± 36	48 ± 7	34 ± 11	94 ± 31
LPC	2.6 ± 0.3	3.70 ± 1.1	548 ± 70	0.2 ± 0.1	19 ± 7	0.2 ± 0.1	0.7 ± 0.1	50 ± 10

## 4. Acidophilic Eukaryotic Diversity, an Ecological Paradox

Besides its extreme physico-chemical water characteristics, what makes Río Tinto a unique acidic environment is the unexpected degree of eukaryotic diversity found in its waters [20,21] and the fact that eukaryotic organisms are the principal contributors of biomass in the habitat (over 65% of the total biomass). Members of the phylum *Chlorophyta* such as *Chlamydomonas*, *Chlorella*, and *Euglena*, are the most frequent species followed by two filamentous algae belonging to the genera *Klebsormidium* and *Zygnemopsis* [24,25]. The most acidic part of the river, is inhabited by a eukaryotic community dominated by two species related to the genera *Dunaliella* and *Cyanidium* (*Rhodophyta*) well known for their high metal and acid tolerance [26]. Pennate diatoms are also present in the river forming large brown biofilms. These biofilms are usually clearly dominated by only one species related to the genus *Pinnularia*. Species belonging to these genera, especially *Pinnularia*, are fairly widespread at environments with pH values around 3.0 [27]. From all the environmental variables that affect freshwater diatoms, pH seems to be the most important and, most taxa show a preference for a narrow pH range [28]. The low diversity of diatoms present in Río Tinto in comparison with the diversity found in neighboring freshwaters, supports the idea that there is a threshold between pH 4.5 and 3.5 in which many species of diatoms are eliminated [27].

Molecular ecology techniques have identified algae closely related to those characterized phenotypically, emphasizing the high degree of eukaryotic diversity existing in the extreme conditions of Río Tinto [20,29]. Within the decomposers, fungi are very abundant and exhibit great diversity, including yeast and filamentous forms. A high percentage of the isolated hyphomycetes are able to grow in the extreme conditions of the river. Some of the isolated yeast species can also be found in less extreme aquatic environments, but the isolated dematiaceae seems to be specific to the extreme conditions of the habitat [30,31].

Additionally, fungal species such as *Hortaea werneckii* and *Acidomyces acidophilum* have been detected in Río Tinto by using molecular techniques [20]. The mixotrophic community is dominated by cercomonads and stramenopiles related to the genus *Bodo*, *Ochromonas*, *Labyrinthula* and *Cercomonas*. The protistan consumer community is characterized by two different species of ciliates tentatively assigned to the genera *Oxytrichia* and *Euplotes*. Amoebas related to the genus *Valhkampfia* and *Naegleria* can be found frequently even at the most acidic parts of the river (pH 1) and one species of heliozoan belonging to the genera *Actinophyris* seems to be the characteristic top predator of the benthic food chain in the river. We know from microscopic observations that rotifers also inhabit the river [21,24].

However, not only unicellular eukaryotic systems develop in the extreme conditions of the Tinto Basin. Different plants can be found growing in the acidic soils of the river banks (pH ca. 3, de la Fuente and Amils, personal communication). The strategies used by these plants to overcome the physiological problems associated to the extreme conditions of the habitat are diverse. Some are resistant to the heavy metals concentrated in the soils in which they grow while others specifically concentrate metals in different plant tissues. Recent analysis by XRD and Mossbauer spectroscopy of the iron minerals found in the rhizomes and leaves of *Imperata cylindrica*, an iron hyperaccumulator perennial grass growing in the Río Tinto banks, showed significant concentrations of jarosite and iron oxyhydroxides [32]. These results suggest that the management of heavy metals, in general, and iron, in particular, is much more complex and versatile in plants than has been reported to date [33]. Also, these results prove that multicellular complex systems can also develop in some extreme conditions, like those existing in Río Tinto.

**Figure 2:**
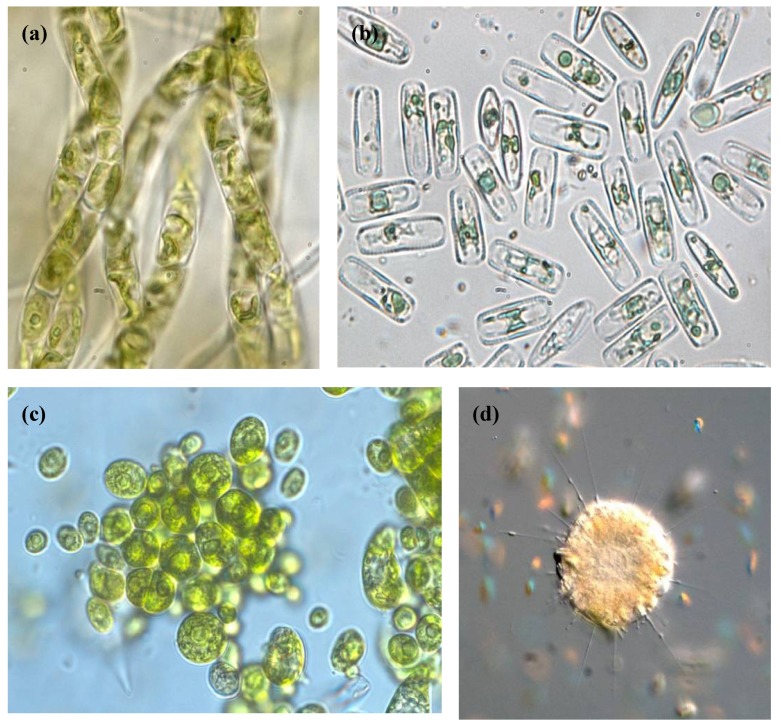
Light microscopy photographs of different eukaryotic species isolated from Río Tinto. (**a**) Filamentous green algae *Klebsormidium* sp.; (**b**) Diatoms; (**c**) Green algae *Chlamydomonas* spp.; (**d**) Heliozoa *Actinophrys* sp.

As previously discussed, the prokaryotic diversity in Río Tinto water column is rather low, which corresponds to what should be expected from an extreme environment. In contrast, the unexpectedly high level of acidophilic eukaryotic diversity (Figure 2) poses an ecological paradox that is not well understood. It is obvious from these observations that adaptation to the extreme conditions of Río Tinto must be much easier than what we thought.

The extreme conditions of this ecosystem are rather recent (2 My) [34], so the adaptation of these complex organisms, which can be found in neutral aquatic environments nearby, to proton gradients between the inner (pH near neutrality) and outer part of the membranes (pH around 2) of five orders of magnitude and high concentrations of very toxic heavy metals (As, Cu, Zn, Cr, Al), must be relatively fast and efficient [29].

## 5. Photosynthesis in Acidic Environments

Photosynthesis is known to be particularly sensitive to stressful environmental conditions, such as salinity, pH or presence of toxicants. There are relatively few reports regarding photosynthesis in acidic environments in the literature, and most have focused on primary productivity measurements in acidic lakes. Thus, it has been reported that minimum primary productivity is mainly due to metal stress [35] or soluble reactive phosphate concentration [36]. However, low pH itself does not reduce photosynthetic activity [37]. Light is another limiting factor for primary productivity in acidic lakes. Adaptation to low light intensities has been reported for benthic biofilms of diatoms in acidic lakes [38].

In Río Tinto, the phototrophic biofilms analyzed exhibiting photoinhibition (*Euglena mutabilis*, *Pinnularia* sp. and *Chlorella* sp.) are usually located at the bottom of the river bed, covered by several centimetres of highly coloured red water. However, *Zygnemopsis* sp. showed a photosaturated behaviour probably because is a filamentous alga usually found at the water surface during the summer, when the sun irradiance is extremely high, and in this way can be considered a high light adapted species. In addition, the analyzed species can be considered as low light or shade adapted organisms due to their low Ic and Ik values (Table 2), which may be related to the fact that they develop under highly colored waters, that affect quantitatively and qualitatively the light available for phototrophic organisms [39]. Even at irradiances as low as 5 μmol m^−2^ s^−1^, in the case of the diatom *Pinnularia* sp., photosynthetic activity was detected. These results are in agreement with previous data from sediments of acidic lakes, where photosynthetic ability at low light intensities (<1.2 μE m^−2^ s^−1^) were found in a benthic biofilm of diatoms suggesting an efficient absorption of red light, the dominant wavelength available in these iron-rich acidic waters, by these organisms [38]. Maximum photosynthesis values (Pmax) were also low in comparison with other environments, in which rates higher than 200 μmol O_2_ mg Chla^−1^ h^−1^ are usually reached [40]. In acidic mining lakes, planktonic primary productivity is usually low probably due to the low phytoplankton biomass [41]. In our case, this cannot be the reason, since in Río Tinto, representing over 65% of the total biomass [30]. Another suggested reason for the low productivity in these extreme environments could be the lack of nutrients such as ammonium, phosphate or nitrate [36].

**Table 2:** Photosynthetic parameters of the different biofilms (*Chlorella*, *Euglena*, Diatom and *Zygnemopsis*) isolated from different locations at Río Tinto (AG, ANG, 3.1, NUR, SM and LPC). Compensation light intensity (Ic) and light saturation parameter (Ik) are expressed on photon basis (μmol photons m^−2^ s^−1^). Photosynthetic efficiency (α) and photoinhibition factor (β) are expressed on Chl a basis (μmol O_2_ mg Chla^−1^ h^−1^) [39].

Species	Ic	Ik	α	β
Chlo_AG	10.36 ± 3.26	59.65 ± 7.03	0.448 ± 0.13	0.0123 ± 0.01
Chlo_ANG	23.19 ± 3.24	120.34 ± 8.08	0.137 ± 0.02	0.0431 ± 0.01
Eug_3.1	18.93 ± 0.72	95.41 ± 9.23	0.448 ± 0.09	0.0450 ± 0.02
Eug_AG	18.44 ± 5.34	49.91 ± 5.89	0.278 ± 0.12	0.0441 ± 0.00
Eug_NUR	16.84 ± 0.76	96.23 ± 5.32	0.263 ± 0.02	0.0247 ± 0.02
Eug_SM	17.43 ± 4.59	48.53 ± 5.32	0.558 ± 0.05	0.0179 ± 0.00
Dia_NUR	5.06 ± 1.72	47.82 ± 6.47	1.423 ± 0.10	0.0426± 0.02
Zyg_LPC	38.89 ± 22.70	13.22 ± 3.23	0.249 ± 0.03	

All the photosynthetic parameters analyzed showed statistical significant differences among species and sampling locations. Thus, the *Euglena* biofilms isolated from different habitats of the Río Tinto (3.1, AG, NUR, and SM) showed different photosynthetic values despite they are mainly formed by the same phototrophic species. These three sampling locations showed different water environmental physochlemical characteristic [39]. These results could be explained by photoadaptation processes instead of photoacclimation procedures. Photoadaptation refers to changes in the genotype that arise either from mutations or from changes in the distribution of alleles within a gene pool, while photoacclimation refers to phenotypic adjustments that arise in response to variations of environmental factors. 

**Figure 3:**
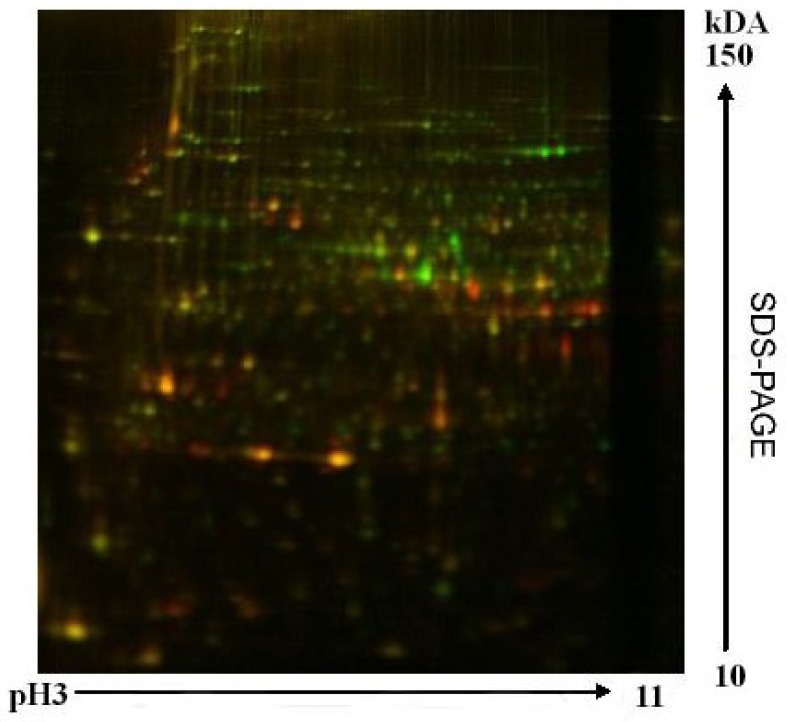
2-DE preparative gels. The spots resolved by 2-DE from preparative gels were stained with (**A**) Cy3 cells growing under BG11/f2 artificial media at pH2. (**B**) Cy5 cells growing under natural metal-rich water NW/f2 at pH2.

Using proteomic analysis of global expression patterns of cellular soluble proteins in an acidophilic strain of *Chlamydomonas* sp. we found that several stress-related proteins are induced in the cells growing in natural river water, along with a complex battery of proteins involved in photosynthesis, primary and energy metabolism or motility [42]. When the 2-DE gels were compared, some of the most dramatic changes observed were related to proteins involved in the Calvin cycle and photosynthetic metabolism. In fact, three of the nine identified downregulated proteins found in cells grown in the presence of metals, were described from these metabolic pathways (Figure 3). The amount of the ribulose-1,5-bisphosphate carboxylase/oxygenase (RuBisCo) decreases significantly when cells grow in metal rich water. This decrease correlates with other proteins described from photosynthesis, such us cytrochrome C peroxidase, oxygen-evolving enhancer protein or photosystem I 11K protein precursor.

These results are closely related to the presence of high levels of heavy metals present in the natural acidic waters since, inhibition of photosynthetic activity is one of the most important cellular responses to metal stress conditions [43,44]. Similar results were found in *C. reinhardtii* in the presence of cadmium and copper [45,46], as well as in other photosynthetic organisms [47,48]. Although growth in extreme acidic environments is expected to require specific cellular adaptations of photosynthetic organisms, other studies have reported stress symptoms in acidophilic *Chlamydomonas* growing under acidic or metal-enriched natural water [36,49]. On the contrary, phytochrome B, phosphoribulokinase and phosphoglycerate kinase were up-regulated when cells were grown in metal rich acidic water. Phytochromes are a family of light-sensing proteins required for plant developmental responses to light [50]. Plants perceive the intensity, direction, and quality of light and use this information to optimize photosynthesis. Phytochrome is the best characterized of the photoreceptors involved in these light dependent responses. In our case, the induction of this protein in the cells under study could be due to the intense red color of Río Tinto water, caused by the high concentration of soluble ferric iron at the low pH of the river. This color has a marked effect on the quality and intensity of the light that reaches the cells. Experiments carried out in acidic mining lakes showed that only red light reaches the sediments of iron-rich water [38]. The increased levels of phytochrome could be an adaptation process to these environmental conditions. The remaining induced enzymes, phosphoglycerate kinase and phosphoribulokinase are similar results were found for *C. reinhardtii* under cadmium exposure suggesting a limitation of the photosynthetic electron transfer that might force the cell to reorganize its whole metabolism [46].

## 6. Conclusions

Extremophiles are not only important resources for developing novel biotechnological processes, but also ideal models for research in the ecological and molecular fields. An understanding of the versatility of life on Earth, as well as the mechanisms that allow some organisms to survive or develop in extreme conditions, will help to gain more insights into the evolution of life, the development of special ecosystems and in the search for life beyond Earth. Although most habitats have not been sufficiently well explored, colonization of extreme habitats is not usually limited to a unique taxonomic domain, and eukaryotic species are exceptionally adaptable comparable in this aspect to the prokaryotes, at least in this acidic environment. Among different extreme environments, acidic habitats are rather peculiar because in most cases they are the product of metabolism of active chemolithotrophic microorganisms. The microbial diversity characterization of Río Tinto allowed for detection of a high level of eukaryotic diversity, which contrasts with the rather low level of prokaryotic diversity found in the system. The highest concentration of biomass of the ecosystem corresponds to photosynthetic algae, although other protists, yeast and filamentous fungi have been isolated along the river. Most the photosynthetic eukaryotes in the Tinto ecosystem are forming biofilms. In this case, the distribution of these photosynthetic communities seems to be more influenced by the presence of heavy metals, than low pH. The high level of eukaryotic diversity found in the Tinto basin demonstrates that complex eukaryotic systems can thrive and dominate extremely acidic, heavy metal-laden environments. Since some of these acidophiles are closely related to cultured neutrophiles, we can conclude that eukaryotes must have the ability to adapt from neutral to acidic environments over relatively short periods of time. Thus, eukaryotic extremophiles are more widely distributed and phylogenetically diverse than previously thought.
